# Combined transcriptomic and metabolomic analysis reveals the potential mechanism of seed germination and young seedling growth in *Tamarix hispida*

**DOI:** 10.1186/s12864-022-08341-x

**Published:** 2022-02-08

**Authors:** Xin’an Pang, Jiangtao Suo, Shuo Liu, Jindong Xu, Tian’ge Yang, Niyan Xiang, Yue Wu, Bojie Lu, Rui Qin, Hong Liu, Jialing Yao

**Affiliations:** 1 College of Life Science and Technology, Huazhong Agricultural University, Wuhan, 430070 Hubei China; 2 Key Laboratory of Protection and Utilization of Biological Resources in Tarim Basin, Xinjiang Production and Construction Corps, College of Life Sciences, Tarim University, Alar, 843300 China; 3 Hubei Provincial Key Laboratory for Protection and Application of Special Plant Germplasm in Wuling Area of China, College of Life Sciences, South-Central University for Nationalities, Wuhan, 430074 Hubei China

**Keywords:** *Tamarix hispida*, Seed germination, Post-germination, Transcriptome, Metabolome

## Abstract

**Background:**

Seed germination is a series of ordered physiological and morphogenetic processes and a critical stage in plant life cycle. *Tamarix hispida* is one of the most salt-tolerant plant species; however, its seed germination has not been analysed using combined transcriptomics and metabolomics.

**Results:**

Transcriptomic sequencing and widely targeted metabolomics were used to detect the transcriptional metabolic profiles of *T. hispida* at different stages of seed germination and young seedling growth. Transcriptomics showed that 46,538 genes were significantly altered throughout the studied development period. Enrichment study revealed that plant hormones, such as auxin, ABA, JA and SA played differential roles at varying stages of seed germination and post-germination. Metabolomics detected 1022 metabolites, with flavonoids accounting for the highest proportion of differential metabolites. Combined analysis indicated that flavonoid biosynthesis in young seedling growth, such as rhoifolin and quercetin, may improve the plant’s adaptative ability to extreme desert environments.

**Conclusions:**

The differential regulation of plant hormones and the accumulation of flavonoids may be important for the seed germination survival of *T. hispida* in response to salt or arid deserts. This study enhanced the understanding of the overall mechanism in seed germination and post-germination. The results provide guidance for the ecological value and young seedling growth of *T. hispida*.

**Supplementary Information:**

The online version contains supplementary material available at 10.1186/s12864-022-08341-x.

## Background

Seed germination and post-germination (early seedling growth) are controlled by various environmental factors and are important stages for the survival of higher plants under ambiently suitable environment [1]. Many physiological and morphological studies have been conducted on related processes, including plant pigment regulation [2–4], abiotic stress [5–8] and plant hormone regulation [9–11]. These stages require a large amount of energy and nutrients which can only be obtained from seed reserves because the germinated seeds cannot absorb minerals and produce energy through photosynthesis [12]. Seed germination begins with the water absorption by the stationary dry seed and is completed by radicle protrusion through the surrounding germ tissues. This process involves a series of orderly physiological and morphogenetic processes, such as energy conversion, nutrient consumption and metabolite changes [13]. After dry seeds have absorbed water, glycolysis, pentose phosphate pathways, and tricarboxylic acid (TCA) cycles are activated. Glycolysis and the TCA cycle provide most of the energy for seed germination [14]. Seed germination and early seedling growth are regulated through a complex network of signalling and gene expression regulation. For example, seed germination can be regulated by multiple plant hormones [15, 16], such as the antagonistic action of abscisic acid (ABA) and gibberellin (GA) [17] and the interaction of jasmonic acid (JA), indole-3-acetic acid (IAA) and other phytohormones [18].

Different plants may have similar molecular mechanisms, including plant hormonal behaviour, transcription and translation activation and radicle protrusion. However, different plant species also have some unique mechanisms, especially for reserve mobilisation and metabolic activation. These differences may be attributed to their different seed stocks [19]. With the rapid development of system biology and high-throughput sequencing, multi-omics technology has become an indispensable research tool in life science [20, 21]. This method can extensively analyse the cell life cycle in all aspects by identifying gene transcripts, metabolites and protein changes throughout growth and development from cell to tissue and the individual itself to understand the complex mechanism of plants and animals.

One of the extremely saline/alkali-tolerant plants, *Tamarix hispida* Willd. is a typical woody halophyte that forms a natural forest in 1% saline–alkali soil of desert environments. This species is also tolerant to drought stress and thus is an ideal material for cloning drought and saline tolerance-related genes and studying the saline tolerance mechanism of woody halophytes [22]. *ThSAP30BP* may play an important physiological role in the salt tolerance of *T. hispida* [23]. The *2-Cys* peroxidase gene of this plant improves its tolerance to salt stress [24]. *ThNAC7* induces the transcriptional levels of genes associated with stress tolerance to enhance salt and osmotic tolerance by increasing osmotic potential and enhanced ROS scavenging capability [25]. *ThMYB13* may also play a role in salt stress tolerance in this transgenic plant [22]. The bZIP protein *Thbzip1* of *T. hispida* is an ACGT elemental binding factor that enhances abiotic stress signalling in transgenic *Arabidopsis thaliana* [26]. However, the molecular mechanisms underlying the seed germination and post-germination of *T. hispida* have not been reported. Transcriptomic sequencing is an effective tool to understand complex molecular regulatory mechanisms and provide a new perspective on *T. hispida* seed germination. At present, this method is routinely used as an experimental platform and has made important contributions to the discovery and identification of genes involved in metabolic pathways [27]. Metabolomics can reveal the terminal products of the signalling pathway and consequently reflect the physiological state of an organism at a specific time. Metabolomes are quite similar to phenotypes and thus can provide detailed information about the intracellular activities regulated by metabolites. Therefore, metabolomics has been widely used in model plants and crops, such as *A. thaliana* [28–30], rice [31–33], soybean [34, 35] and many other medical plants [36–38].

In this study, the mechanisms underlying the germination and post-germination of *T. hispida* were investigated using an integrative transcriptomic and metabolomic approach. Gene Ontology (GO) enrichment analysis of differentially expressed genes (DEGs) indicated the differential involvement of biological processes in the six stages of seed germination and post-germination. The dynamic changes of the key genes in flavonoid biosynthesis and phytohormone-related were observed also observed. The Combined transcriptomic and metabolomic analysis showed the accumulation of flavonoids in post-germination. The results provide a valuable reference for the study of seed germination and its functions and effects on saline/alkali-tolerant plants.

## Results

### Stage definition and transcript assembly of seed germination and post-germination in *Tamarix hispida*

**Fig. 1:**
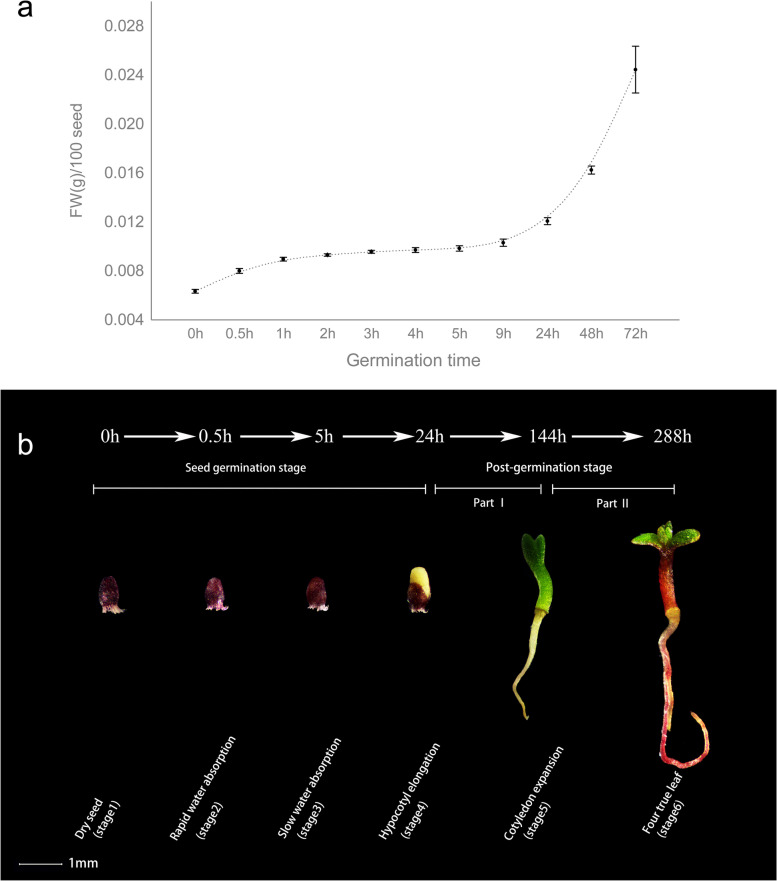
Schematic of the seed germination and fresh weight curve of water absorption in *Tamarix hispida*. **a** Curve of fresh weight of seeds with time after water absorption. **b** Representation of morphological changes in seed germination and young seedling growth

Seed germination can be divided into the following three stages according to Bewley’s definition [12]: rapid water imbibition, limited water absorption and increase in water uptake accompanied by embryonic axis elongation. For *T. hispida*, the dry seeds were labelled as stage 1 at 0 h, followed by the rapid increase in water uptake at 0.5 h as stage 2, a slow increase in water uptake at 5 h as stage 3 and hypocotyl extension period at 24 h as stage 4 **(**Fig. 1a and Additional file 1: Table S1). Post-germination comprised stage 5 with cotyledon unfolding at 144 h and stage 6 with four-true-leaf unfolding at 288 h (Fig. 1b). All these defined stages are shown in Fig. 1b.

Eighteen samples from six stages (1–6, three replicates for each stage) were sampled for RNA-seq. A total of 866,487,598 high-quality reads with Q30 higher than 90% were obtained after quality control, and 75,249 unigenes were de novo assembled using Trinity software. The length was over 1000 bp for 36,531 unigenes (48.6%) and over 1500 bp for 23,089 (30.1%) unigenes. The average gene length and N50 length were 1502 and 2031 bp, respectively. Functional unigene annotation was performed and mapped to NCBI non-redundant (Nr) (48,606, 64.6%), eggNOG database (39,772, 52.9%), Swiss-Prot (33,428, 44.4%), Kyoto Encyclopedia of Genes and Genomes (KEGG) (12,510, 16.6%) and GO (26,371, 35%) (Additional file 2: Fig. S1). A summary of RNA-seq data is shown in Table 1.

**Table 1:** Statistics and functional annotations of unigenes in 18 RNA sequencing libraries

Feature	Number of unigenes	Percentage (%)
≥ 1000 bp	36,531	48.5
≥ 1500 bp	23,089	30.7
≥ 2000 bp	16,012	21.3
N50	2031(bp)	–
Max length	19,692(bp)	–
Min length	501(bp)	–
Average length	1502(bp)	–
NR	48,606	64.6
EggNOG	39,772	52.9
SwissProt	33,428	44.4
KEGG	12,510	16.6
GO	26,371	35.0
Total	75,249	100.0

### Differential expression and functions of the genes involved in seed germination and post-germination

**Fig. 2:**
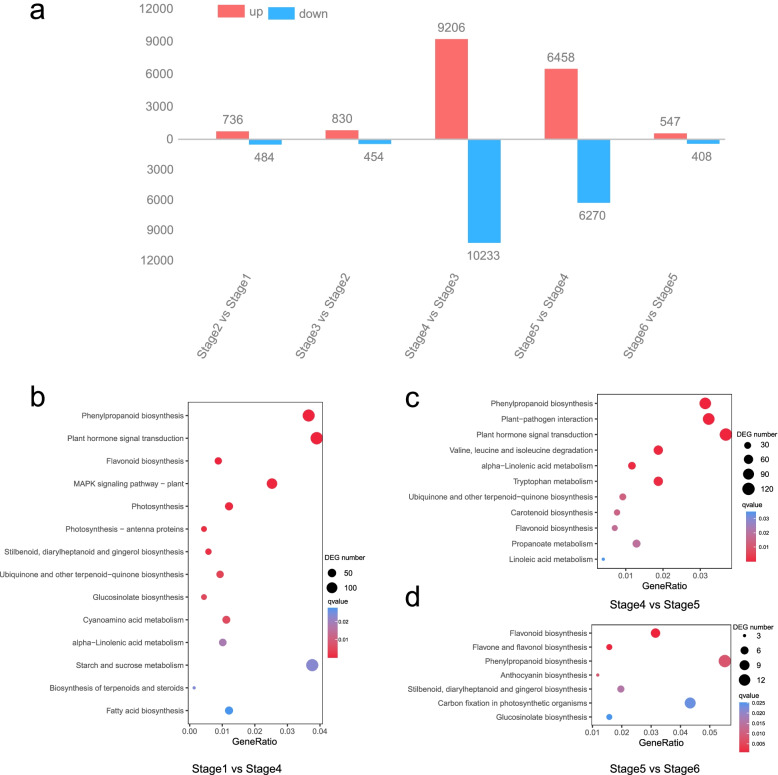
Differential expressed genes (DEGs) during the six stages of *Tamarix hispida* seed germination and post-germination. **a** Numbers of up-regulated and down-regulated DEGs at adjacent stages. Enriched DEGs from Kyoto Encyclopedia of Genes and Genomes (KEGG) pathways. **b** Enriched KEGG pathways of DEGs between stages 1 and 4. **c** Enriched KEGG pathways of DEGs between stages 4 and 5. **d** Enriched KEGG pathways of DEGs between stages 5 and 6

Pairwise differential expression profiling analysis was conducted with the threshold of FDR ≤ 0.05 and absolute value fold change ≥ 2.0 using DEseq2 software to investigate the molecular basis of seed germination and post-germination [39]. Various numbers of DEGs among neighbouring stages were identified as follows: 1220 between stages 1 and 2 (736 up, 484 down), 1284 between stages 2 and 3 (830 up, 454 down), 19,439 between stages 3 and 4 (9206 up, 10,233 down), 12,728 between stages 4 and 5 (6458 up, 6270 down) and 955 between stages 5 and 6 (547 up, 408 down) (Fig. 2a, Additional file 1: Table S2). This result showed that the number of DEGs in the hypocotyl extension period (stage 3 versus stage 4) of seed germination and the cotyledon unfolding period (stage 4 vs. stage 5) of seed post-germination was higher than that in the other comparison groups.

GO and KEGG enrichment analysis for adjacent compared groups reflected the physiological response of germination and post-germination. For example, the most enriched DEGs were identified in the GO term of ‘cellular response to osmotic stress’ in the comparison between stages 1 and 2. Meanwhile, the most enriched DEGs were observed in the KEGG pathway of ‘metabolism of xenobiotics by cytochrome P450’ in the comparison between stages 1 and 2, indicating that cytochrome P450 may be important for the rapid increase in water uptake (Additional file 2: Fig. S2 and S3). Given that seed germination occurs from stage 1 to stage 4, further KEGG enrichment analysis was performed on 18,975 DEGs (stage 1 vs. stage 4). Among these enriched pathways, ‘phenylpropanoid biosynthesis’ (ko00940), ‘plant hormone signal transduction’ (ko04075) and ‘flavonoid biosynthesis’ (ko00941) were the top three significantly enriched categories (Fig. 2b, Additional file 1: Table S3) and thus have important roles in the seed germination of *T. hispida*. At early young seedling during post-germination, ‘phenylpropanoid biosynthesis’ and ‘flavonoid biosynthesis’ were the most significantly enriched in the periods of cotyledon unfolding (part I, stage 4 vs. stage 5) and true-leaf unfolding (part II, stage 5 vs. stage 6). Among the separate top five enriched pathways, ‘plant–pathogen interaction’ and ‘plant hormone signal transduction’ were significantly enriched in part I, and ‘flavone and flavonol biosynthesis’ and ‘anthocyanin biosynthesis’ were significantly enriched in part II (Fig. 2c and d, Additional file 1: Table S3). The enrichment of ‘anthocyanin biosynthesis’ term in true-leaf unfolding period indicated anthocyanin accumulation, which was consistent with the morphology of extremely dark red stem and root compared with those during cotyledon unfolding **(**Figs. 1b and 2d). These results showed that hormone signal transduction and flavonoid biosynthesis may be important for the seed germination and post-germination of *T. hispida*.

### Dynamic regulation of key gene families in flavonoid biosynthesis during seed germination and post-germination

**Fig. 3:**
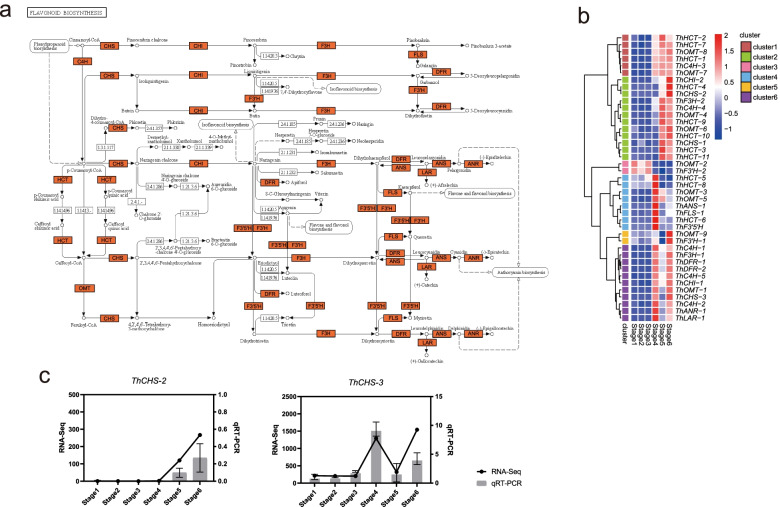
Analysis of DEGs in flavonoid biosynthesis pathway. **a** Mapping of enriched DEGs in flavonoid biosynthesis pathway (ko00941) [42]. The orange marks represent DEG enrichment. **b** Expression pattern of DEGs involved in flavonoid biosynthesis at different stages. **c** FPKM from RNA-seq and expression level from qRT-PCR of two chalcone synthase (CHS) genes. The expression at stage 1 was set as 1, and the relative expression level was calculated for several genes

The phenylpropanoid pathway is the upstream branch of flavonoid biosynthesis; hence, DEGs involved in phenylpropanoid biosynthesis (ko00940) and flavonoid biosynthesis (ko00941) were identified during the seed germination and post-germination of *T. hispida*. A total of 158 DEGs in phenylpropanoid pathway, such as genes encoding phenylalaninammo-nialyase (PAL), 4-coumarate-CoA ligase (4CL) and trans-cinnamate 4-monooxygenase (CYP73A), were overall up-regulated after stage 3 (Additional file 2: Fig. S4). Flavonoid biosynthetic genes were divided into two categories, namely, early biosynthetic genes (EBGs) responsible for the production of common precursors and late biosynthetic genes (LBGs) for the eventual products. The former mainly encodes naringenin-chalcone synthase (CHS), chalcone isomerase (CHI), naringenin 3-dioxygenase (F3H), flavonoid 3′-monooxygenase (F3’H) and flavonoid 3′,5′-hydroxylase (F3’5’H), and the latter mainly encodes flavonol synthase (FLS), dihydrokaempferol 4-reductase (DFR), anthocyanidin synthase (ANS), anthocyanidin reductase (ANR) and leucoanthocyanidin reductase (LAR) [40, 41]. Finally, 41 DEGs were identified in flavonoid biosynthesis pathway, including 35 EBGs and six LBGs (Fig. 3a, Additional file 1: Table S4). A clustering heatmap of gene expression divided these DEGs into six clusters. Except *ThOMT-2* and *ThF3’H-2* of cluster3, all of these genes were up-regulated upon hypocotyl extension. The EBGs of flavonoid biosynthesis were distributed in all clusters, and the LBGs were significantly enriched in cluster6 (*p*-value = 0.035) (Fig. 3b). The cluster4 genes were upregulated in stage 3, and the cluster6 genes were upregulated in stages 4 and 6. Further validation of *ThCHS2* (*TRINITY_DN7978_c0_g1*) of cluster4 and *ThCHS3* (*TRINITY_DN8_c1_g1*) of cluster6 by real-time quantitative reverse transcriptional PCR (qRT-PCR) experiment indicated the differential regulation of CHS genes **(**Fig. 3c**)**. Compared with those in seed germination, more genes were involved in the phenylpropanoid and flavonoid biosynthesis pathways of post-germination and further contributed to flavonoid accumulation. This high level of flavonoid would help young seedlings to survive from extreme drought desert environments.

### Dynamic regulation of phytohormone-related DEGs during seed germination and post-germination

**Fig. 4:**
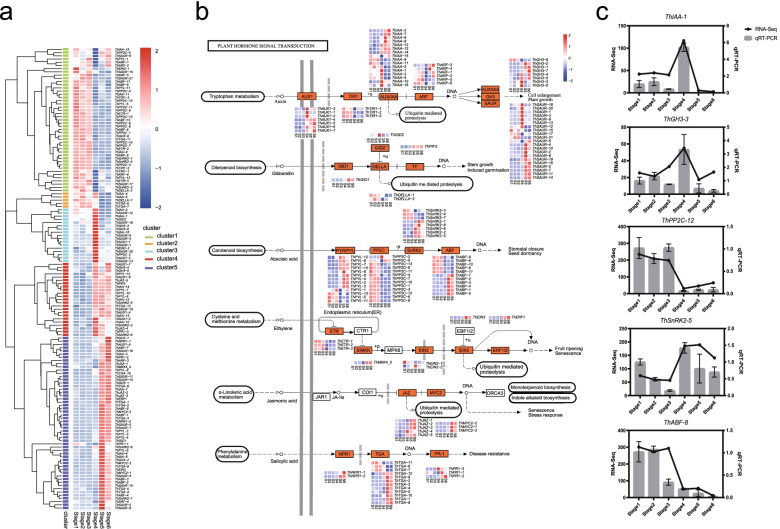
Analysis of DEGs in plant hormone signal transduction **a** Heatmap of DEG expression of auxin, gibberellin (GA), abscisic acid (ABA), ethylene (ETH), jasmonic acid (JA) and salicylic acid (SA) signalling pathways at different stages. **b** DEG expression enriched in auxin, GA, ABA, ETH, JA and SA signalling pathways [42]; expression levels are indicated by the heatmap for different groups. The orange marks represent DEG enrichment. **c** FPKM from RNA-seq and expression level from qRT-PCR of phytohormone-related DEGs. The expression at stage 1 was set as 1, and the relative expression level was calculated for several genes

A total of 144 DEGs were significantly enriched in ‘plant hormone signal transduction’ (ko04075) in seed germination and post-germination, suggesting their important roles in the seed germination of *T. hispida*. Further classification of these hormone-related DEGs showed that 61 were involved in auxin (AUX) pathway, five in GA pathway, 45 in ABA pathway, eight in ethylene (ETH) pathway, eight in JA pathway and 17 in salicylic acid (SA) pathway (Additional file 1: Table S5). The 144 phytohormone-related DEGs were further divided into five expression clusters. Among which, cluster1 genes were upregulated in stages 1–3, cluster2 genes in stages 1–4, cluster3 genes only in stage 4, cluster4 genes in stages 4–6, and cluster5 genes in stages 5 and 6 (Fig. 4a).

The DEGs (14/61) of AUX pathway were significantly enriched in cluster3 (17 genes) (*p*-value = 0.0005) and upregulated in stage 4, indicating the important role of auxin signalling in hypocotyl elongation. The DEGs (24/45) of ABA pathway were significantly enriched in cluster1 (45 genes) (*p*-value = 0.00016) and consistently upregulated in stage 1–3, implying the regulatory function of ABA in seed germination such as water uptake. The JA (6/8) and SA (12/17) genes were enriched in cluster5 (54 genes) (JA: p-value = 0.032, SA: *p*-value = 0.0035) and upregulated in stages 5 and 6 of post-germination. For example, jasmonate ZIM domain-containing protein (JAZ), NONEXPRESSER OF PR GENES 1 (NPR1) and transcription factor TGA (TGA) were significantly up-regulated during cotyledon expansion and true-leaf unfolding. The expression patterns of the identified phytohormone-related DEGs were mapped in plant hormone signal transduction (Fig. 4b). Five genes [*IAA* (*TRINITY_DN37691_c0_g1*), auxin responsive *GH3* gene family (*TRINITY_DN38209_c0_g1*), *PP2C* (*TRINITY_DN4920_c0_g1*), serine/threonine-protein kinase *SnRK2* (*TRINITY_DN1976_c0_g1*) and *ABF* (*TRINITY_DN7836_c2_g100)*] were randomly selected for qRT-PCR confirmation to further validate their differential transcription (Fig. 4c). The results suggested that different plant hormones play varying roles in the seed germination and post-germination of *T. hispida*.

### Co-expression network analysis with WGCNA

**Fig. 5:**
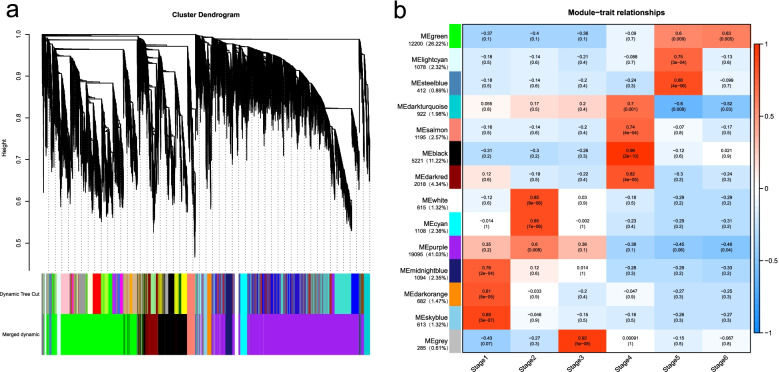
Weighted gene co-expression network analysis (WGCNA) of genes during the seed germination and young seedling growth of *Tamarix hispida*. **a** Dendrogram showing the co-expression modules identified by WGCNA across seed germination and post-germination. The major tree branches constitute 14 modules labelled with different colours. **b** Module-stage association (each row corresponds to a module, and each column represents a specific stage. The colour of each cell at the row-column intersection indicates the correlation coefficient between a module and the stage). Red indicates the positive correlation between the module and the stage, and blue indicates a negative correlation

A global co-expression network with all DEGs (46,538 genes) was constructed by weighted gene co-expression network analysis (WGCNA) to identify additional potential genes involved in seed germination and post-gemination. Thirteen co-expressed modules were obtained on the basis of the network concept (Fig. 5a), and stage-associated modules were identified by correlation analysis between module eigengenes and each stage (Fig. 5b). Among the positive stage-correlated modules with Pearson correlation coefficient (PCC) ≥ 0.60, three modules of ‘midnightblue’, ‘darkorange’ and ‘skyblue’ were correlated with stage 1; three modules of ‘white’, ‘cyan’ and ‘purple’ with stage 2; four modules of ‘darkturquoise’, ‘salmon’, ‘black’ and ‘darkred’ with stage 4; three modules of ‘green’, ‘lightcyan’ and ‘steelblue’ with stage 5; and one module of ‘green’ with stage 6. Except for an invalid ‘grey’, no module was correlated with stage 3 (Fig. 5b). GO and KEGG enrichment analyses of the gene sets merged from specific stage-associated modules were performed to further investigate the biological processes or pathways of the stage-associated modules. Different processes or pathways were enriched in germination and post-germination stages. In seed germination, ‘transposition, RNA-mediated’ GO term, and ‘apoptosis’ KEGG pathway were enriched in stage 2. Multiple GO processes, such as ‘translation’, ‘tricarboxylic acid cycle’ and ‘chloroplast organisation’ were enriched in stage 4. GO terms of ‘carbamoyl-phosphate synthase (glutamine-hydrolysing) activity’ and ‘protein autophosphorylation’ were the most enriched in stages 5 and 6, respectively. (Additional file 2: Fig. S5, Additional file 1: Table S6). Therefore, the co-expressed genes involved in multiple biological processes during stages 2 and 4 were essential for the seed germination and early seeding growth of *T. hispida*.

**Fig. 6:**
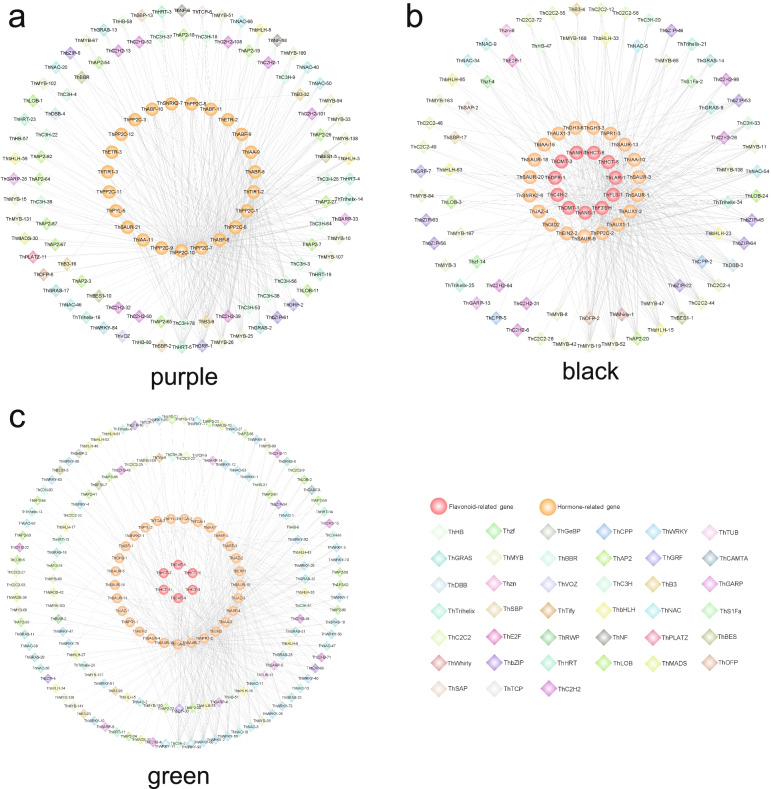
Regulatory network of key phytohormones and flavonoid metabolites. Regulatory network of purple, black and green modules shown in **a**, **b** and **c**, respectively. The orange circles represent structural genes involved in plant hormone metabolism, the red circles represent structural genes involved in flavonoid metabolism, and the diamonds with different colours represent different families of transcription factors identified in the same module whose transcripts are correlated with the expression of structural genes

Genes from three modules (‘purple’, ‘black’ and ‘green’) were further identified for transcriptomic analysis and visualised to understand the possible regulatory mechanism in the key developmental stages of seed germination and post-germination. GO enrichment analysis of the genes of ‘purple’ module revealed multiple terms related to phytohormone response and abiotic stress response, such as ‘response to heat’, ‘response to auxin’ and ‘response to abscisic acid’ (Additional file 2: Fig. S6, Additional file 1: Table S6). The node gene with a high connectivity within a module is defined as hub gene with an important role in different modules. In the ‘purple’ module, various transcription factors (TFs) with high connectivity were involved in hormone response and abiotic stress response. For example, *DEHYDRATION-RESPONSIVE ELEMENT BINDING PROTEIN 2A* (*DREB2A*, *ThAP2–7*) was involved in seed germination and induced by drought and osmotic stress [43], and *DIVARICATA2* (*DIV2*, *ThMYB-138*) was required for ABA signalling and response to salt stress in *Arabidopsis* [44] (Fig. 6a). The ‘black’ module had the highest correlation with hypocotyl elongation (stage 4). Function analysis of high connectivity TFs showed that the hub genes of ‘black’ module were connected with plant organ development and flavonoid biosynthesis. For example, *TRANSPARENT TESTA 2* (*TT2*, *ThMYB-52*) positively regulated anthocyanin accumulation in hypocotyls [45] and *YABBY1* (*YAB1*, *ThC2C2–44*) and *YABBY5* (*YAB5*, *ThC2C2–4*) controlled leaf blade development [46, 47] (Fig. 6b, Additional file 1: Table S6). Meanwhile, ‘phenylpropanoid biosynthesis’, ‘plant hormone signal transduction’ and ‘flavonoid biosynthesis’ were significantly enriched in ‘green’ module (Additional file 2: Fig. S6, Additional file 1: Table S6). Several genes in ‘green’ module encoded by WRKY DNA-BINDING PROTEIN (WRKY) TFs with high connectivity, such as *WRKY DNA-BINDING PROTEIN 42* (*WRKY42*, *ThWRKY-93*), *WRKY DNA-BINDING PROTEIN 33* (*WRKY33*, *ThWRKY-2* and *ThWRKY-70*) and *WRKY DNA-binding protein 4* (*WRKY4*, *ThWRKY-56*), were related to response to abiotic stress; their homologs are also involved in response to abiotic stress in *Arabidopsis* [48–50] (Fig. 6c).

### Combination of metabolic profiling and transcriptomic analysis

**Fig. 7:**
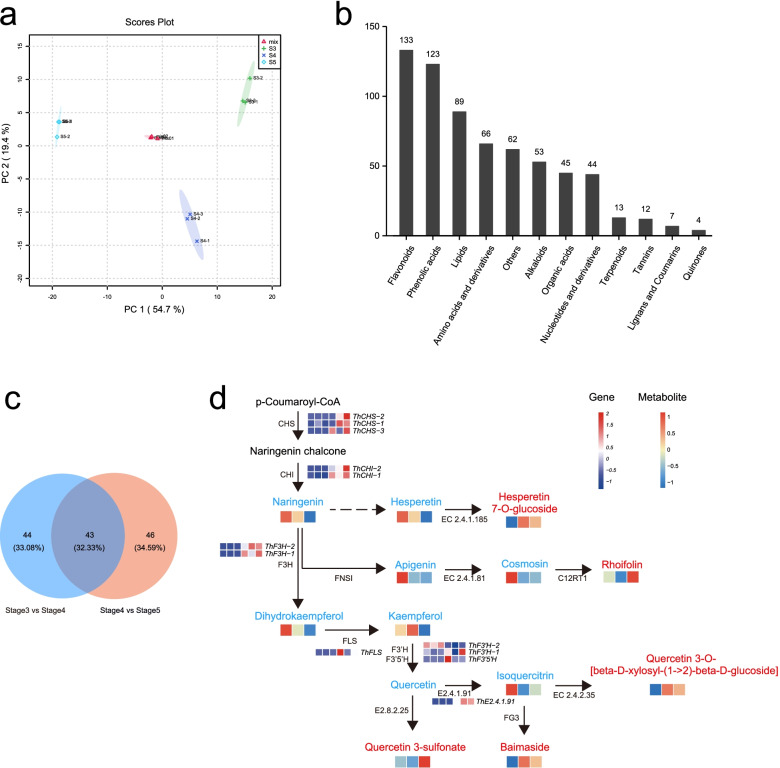
Metabolome analysis of *Tamarix hispida*. **a** Principal component analysis (PCA) of metabolome data in stages 3, 4 and 5. **b** Statistics of different metabolites in stage 3 vs. stage 4 and stage 4 vs. stage 5 (VIP > 1, |log2(FC)| > 1). **c** Venn diagram of flavonoids in differential metabolites. **d** Schematic of a portion of flavonoid biosynthesis pathway in *Tamarix hispida*. Metabolic intermediates are marked in blue, and end products are marked in red. Heat maps show the transcriptional level of enzymes in the six stages and metabolite level in the three stages (3, 4 and 5). Gene sale bar corresponds to the range of relative transcriptional level of enzymes, and metabolite sale bar corresponds to the range of relative metabolite level

Many flavonoid-biosynthesis related genes were activated and enhanced in post-germination (Fig. 3). A widely targeted metabolomics analysis was performed on three stages, namely, slow water absorption (stage 3), hypocotyl elongation (stage 4) and cotyledon expansion (stage 5) to further understand the metabolic profiles in post-germination. The samples from three stages showed good triplications according to their principal component analysis (PCA) score plots, indicating that metabolite accumulation is stage-specific (Fig. 7a). A total of 1022 metabolites were identified in all samples. Within the threshold of variable importance in projection (VIP) > 1 and absolute value fold change > 2, the numbers of up-regulated and down-regulated metabolites in stage 3 versus stage 4 and stage 4 versus stage 5 were 205 and 149, 287 and 178, respectively. A total of 651 differentially expressed metabolites (DEMs) were identified and mainly comprised of flavonoids (133), phenolic acids (123), lipids (89), amino acids and derivatives (66), alkaloids (53), organic acids (45) and others (Fig. 7b, Additional file 1: Table S7). As the most abundant differentiated metabolite, the accumulation of flavonoid was consistent with the results of gene expression and functional enrichment in flavonoid biosynthesis during seed germination and post-germination.

A combined transcriptomic and metabolomic analysis was performed in stages 3, 4 and 5 to investigate the potential regulation of metabolites. The results showed significantly different accumulation for 43 flavonoids in stage 3 versus stage 4 and stage 4 versus stage 5, 44 flavonoids in stage 3 versus stage 4 and 46 flavonoids in stage 4 versus stage 5 (Fig. 7c). With these identified differential flavonoids, the metabolic intermediates and end products of flavonoid biosynthesis pathway were mapped to a known KEGG pathway. The overall trends of flavonoid biosynthesis suggested a decrease in metabolic intermediates (such as naringenin, dihydrokaempferol, apigenin and hesperetin) but an increase in end products (such as quercetin 3-sulfonate, rhoifolin and baimaside) in seed germination and post-germination (Fig. 7d). The accumulation of end flavonoids in post-germination revealed the consistent upregulation of CHS gene regulation starting at stage 4 (Fig. 3), further indicating that chalcone synthase is a key and limited enzyme for flavonoid biosynthesis in the seed germination of *T. hispida*. During seed germination (stages 3 and 4), the metabolites located downstream of the flavonoid synthesis pathway, specifically hesperetin7-O-glucoside, quercetin 3-O-[beta-D-xylosyl-(1- > 2)-beta-D-glucoside] and baimaside, were significantly increased. Meanwhile, the metabolites located upstream of the flavonoid synthesis pathway, such as naringenin, dihydrokaempferol and isoquercitrin, were significantly decreased (Fig. 7d, Additional file 2: Fig. S7). The metabolite flow from naringenin to quercetin 3-sulfonate also confirms the increase and decrease in the metabolites located upstream and downstream of the flavonoid biosynthesis, respectively, in young seedling growth (stages 4 and 5). These results suggest that the metabolites located downstream of the flavonoid synthesis pathway are beneficial to seed germination and young seedling growth.

## Discussion

*T. hispida* is a perennial shrub or small tree and a woody halophyte that serves as an excellent model for studies on resistance to abiotic stress. Seeds are at the crucial stage of biodiversity and agriculture for plant survival. If environmental conditions are only suitable for plant growth but not for seed germination and young seedling growth, then plants cannot survive in the community [51]. Therefore, the molecular mechanism of seed germination and young seedling growth in *T. hispida* must be explored to understand its adaptative evolution in extremely arid deserts.

Shortening the breeding cycle can help to meet the demand for the seedling supply of *T. hispida*. Transcriptomic and metabolomic analyses were performed to detect the changes in RNA level and metabolome levels during seed germination and young seedling growth. Differential transcriptome expression analysis revealed that the DEGs during seed germination and young seedling growth were significantly enriched in phenylpropanoid biosynthesis, plant hormone signal transduction and flavonoid biosynthesis pathway. Meanwhile, four-true-leaf turning red and anthocyanin biosynthesis pathway were significantly enriched for *T. hispida* seedlings. Flavones are synthesised by the flavonoid pathway, the downstream of phenylpropanoid biosynthesis [52]. These compounds are important in plant defence against biotic and abiotic stresses, such as oxidative damage [53] and UV stress [54]. Therefore, the DEGs and metabolites of flavonoid biosynthesis play an important role in the seed germination and young seedling growth of *T. hispida*.

In this study, the flavonoid biosynthesis genes were divided into six expression patterns. CHS is the first committed enzyme in the conserved flavonoid synthesis pathway [55], and three *ThCHSs* were identified in *T. hispida*. Clustering heatmap showed that two CHSs (*ThCHS1* and *ThCHS2*) were highly expressed in stages 5 and 6, respectively, and *ThCHS3* showed significantly increased expression, albeit in various levels, in stages 4 and 6. The expression model of *ThCHSs* is consistent with flavonoid biosynthesis in *T. hispida*, indicating that flavonoids are produced in large quantities during seed germination and young seedling growth. Meanwhile, the early and late genes of flavonoid biosynthesis pathway showed different expression trends. *ThANR-1*, *ThLAR-1*, *ThDFR-1* and *ThDFR-2* are the downstream genes of flavonoid biosynthesis pathway and were highly expressed in stages 4 and 6. However, some of the early genes of flavonoid biosynthesis pathway such as *ThCHS-1*, *ThCHS-2*, *ThCHI-2* and *ThF3H-2* were not expressed in stage 4. Therefore, the expression of flavonoid biosynthesis DEGs undergoes complex regulation during seed germination and young seedling growth.

Seed germination and young seedling growth are important processes affecting crop production and are influenced by a range of factors, including plant hormones [15].. The most important plant hormones for seed germination are ABA and GA, which have inhibitory and stimulatory effects on seed germination, respectively. In this study, ABA and GA expression significantly varied in seed germination and young seedling growth. For example, *ThABFs* and *ThPP2Cs* were down-regulated in seed germination but up- regulated in young seedling growth. Meanwhile, *ThPIF* and *ThGID2* were up- regulated in seed germination. These findings indicate that ABA and GA play important roles in seed germination and young seedling growth. Auxin by itself is not a necessary hormone for seed germination but can interact with other hormones to affect seed germination and young seedling growth. For example, the release of ARF from repression by miRNA also affects ABA sensitivity during young seedling growth [56]. In addition, the DEGs of auxin pathway are vital for young seedling growth. As stress-response hormones, the upregulation of JA and SA pathways can be helpful to flavonoid accumulation during post-germination [57].

The ‘purple’ module was the largest module in WGCNA (19,095 genes, 41.03%) and was the most relevant to seed water uptake (stage 1: dry seed, stage 2: rapid water absorption, stage 3: slow water absorption). In this module, phytohormone and abiotic stress response related terms were significantly enriched, thereby supporting the importance of phytohormone in seed germination. The ‘green’ module (12,200 genes, 26.22%) is the second largest module, and its genes were positively relevant to post-germination (stage 5: cotyledon expansion, stage 6: four true leaf). Enrichment analysis showed that ‘phenylpropanoid biosynthesis’, ‘flavonoid biosynthesis’ and ‘plant hormone signal transduction’ were significantly enriched in this module, indicating that flavonoid is important to post-germination. As a transition node from seed germination to seedling development, hypocotyl elongation (stage 4) was significantly associated with four modules (‘darkred’, ‘black’, ‘salmon’ and ‘darkturquoise’). Among these four modules, ‘black’ (5221 genes, 11.22%) was the largest and the most relevant. ‘Phenylpropanoid biosynthesis’ and ‘flavonoid biosynthesis’ were also significantly enriched in this module, and its high connectivity node functional TFs were associated with plant organ development and flavonoid biosynthesis. These results further support the importance of flavonoids and phytohormones.

## Conclusions

In this study, the molecular regulation of seed germination and post-germination in *T. hispida* was investigated using an integrated transcriptional and metabolomic method. GO and KEGG analysis showed that the pathways of plant hormone signal transduction, phenylpropanoid biosynthesis, and flavonoid biosynthesis were significantly enriched and thus have important roles in the two developmental periods. The gene families involved in the plant hormone pathway that was enriched in different expression clusters showed differential regulation in seed germination and post-germination, such as ABA for early germination stages, auxin for hypocotyl elongation, JA for cotyledon expansion and SA for four-true-leaf unfolding. Metabolomics showed that the final products of flavonoids such as 3 − O−[beta−D − xylosyl−(1− > 2) − beta−D − glucoside], quercetin, baimaside, rhoifolin, hesperetin− 7 − O − glucoside and quercetin− 3 − O − sulfonate accumulated in post-germination. RNA-seq and metabolomic analysis indicated the importance of flavonoid biosynthesis pathways and identified CHS as the key enzyme for the accumulation of final flavonoid products in post-germination. In addition, organic acids were involved in seed germination. All these results provide important insights into the cellular and metabolic changes underlying *T. hispida* seed germination and young seedling growth.

## Materials and methods

### Plant materials, experimental conditions and fresh weight measurements

The seeds of *T. hispida* were obtained from Alar City, Xinjiang Uygur Autonomous Region of China. The hairs of selected seeds were removed, and germination test was carried out in three replicates (100 seeds per replicate). The seeds were incubated in 3 mL of ultrapure water on two sheets of absorbent paper in a covered glass petri dish at 25 °C. At ambient temperature of 25 ± 1 °C, 100 germinated seeds (± 0.0001 g) were weighed every 15 min at the stage of rapid water absorption between 0 and 1 h. After sampling at a specific time, the excess water was immediately sucked up with absorbent paper, and the samples were quickly frozen in liquid nitrogen and stored at − 80 °C in a refrigerator for transcriptomic and metabolomic analyses. We declare that the research programme complies with relevant institutional, national and international guidelines and legislation, and we have permission to collect *T. hispida* seeds.

### RNA isolation and cDNA library construction for RNA sequencing

Total RNA of samples was isolated and purified using Trizol reagent (Invitrogen, Carlsbad, CA, USA) following the manufacturer’s procedure. The RNA was purified and quantified using NanoDrop 2000 (NanoDrop, Wilmington, DE, USA), and its integrity was assessed by Agilent 2100 with RIN number > 7.0. Total RNA was used to construction the RNA-seq libraries: mRNA was enriched from the total RNA using oligo (dT) magnetic beads, and the final average insert size for the final cDNA library was 350 bp (± 50 bp). Paired-end sequencing was performed on an Illumina Hiseq X-Ten (LC Bio, China) following the vendor’s recommended protocol.

Cutadapt [58] was used to remove the reads containing adaptor contamination, low quality bases and undetermined bases. Sequence quality was then verified using FastQC (http://www.bioinformatics.babraham.ac.uk/projects/fastqc/). After the adaptor and low-quality sequences were removed, the clean reads were assembled into expressed sequence tag clusters (contigs) and de novo assembled into transcripts by using Trinity [59] in the paired-end method. On the basis of the similarity and length of the sequence, the longest transcript was selected as a single gene for subsequent analysis.

### Functional annotation of DEGs

The function of the unigenes was annotated by alignment with the NCBI non-redundant (NR) and SwissProt databases using Blastx (v2.10.1) with a threshold e-value of 10^− 5^. The proteins with the highest hits to the unigenes were used to assign functional annotations. With SwissProt annotation, GO classification was performed by mapping the relation between SwissProt and GO terms. The unigenes were mapped to the KEGG database to annotate their potential metabolic pathways [42, 60, 61] using eggNOG-mapper (v2.0.1–14) [62].

All clean reads were aligned onto pomegranate genome. The trimmed mean of M values (TMM) method was used to calculate the unigene expression abundance. DEGs were detected by DESeq2 (v1.22.2) [39] with the absolute value of log2(fold change) value > 1 and a false discovery rate (FDR) < 0.05 as selection criteria. GO enrichment and KEGG pathway enrichment analyses of DEGs were performed using R (v4.0.3) package clusterProfiler (v3.18.1) [63].

### Weighted correlation network analysis and gene network visualisation

Co-expression networks were constructed using the WGCNA (v1.69) package in R (v3.6.1) [64] with the soft threshold = 12 and the minModuleSize = 400. Eigengene values were calculated for each module and used to test associations with each germination stage. Networks were visualised using Cytoscape v.3.8.2 [65].

### Metabolomic analysis

The sample preparation, extract analysis, metabolite identification, and quantification were performed at Wuhan Metware Biotechnology Co., Ltd. (Wuhan, China) (http://www.metware.cn/) following their standard procedures [66, 67].

Biological samples were freeze-dried by vacuum freeze-dryer (Scientz-100F) and then crushed using a mixer mill (MM 400, Retsch) with a zirconia bead for 1.5 min at 30 Hz. Briefly, 100 mg of lyophilised powder was dissolved in 1.2 mL of 70% methanol solution and vortexed for 30 s for every 30 min for six times in total. The sample was placed in a refrigerator at 4 °C overnight. Following centrifugation at 12,000 rpm for 10 min, the extracts were filtrated (SCAA-104, 0.22 μm pore size; ANPEL, Shanghai, China, http://www.anpel.com.cn/) for UPLC-MS/MS analysis.

The sample extracts were analysed using an UPLC-ESI-MS/MS system (UPLC, SHIMADZU Nexera X2, https://www.shimadzu.com.cn/; MS, Applied Biosystems 4500 Q TRAP, https://www.thermofisher.cn/cn/zh/home/brands/applied-biosystems.html) under the following analytical conditions: UPLC column, Agilent SB-C18 (1.8 μm, 2.1 mm * 100 mm); and mobile phase, solvent A of pure water with 0.1% formic acid and solvent B of acetonitrile with 0.1% formic acid. Sample measurements were performed with a gradient program as follows: initial gradient of 95% A, 5% B; a linear gradient to 5% A within 9 min; 5% A, 95% B for 1 min; and adjusted 95% A, 5.0% B within 1.1 min and kept for 2.9 min. The flow velocity was set as 0.35 mL per minute, the column oven was set to 40 °C, and the injection volume was 4 μL. The effluent was alternatively connected to an ESI-triple quadrupole-linear ion trap (QTRAP)-MS.

LIT and triple quadrupole (QQQ) scans were acquired on a triple quadrupole-linear ion trap mass spectrometer (Q TRAP), AB4500 Q TRAP UPLC/MS/MS System, equipped with an ESI Turbo Ion-Spray interface, operating in positive and negative ion modes and controlled by Analyst 1.6.3 software (AB Sciex). The ESI source operation parameters were as follows: ion source, turbo spray; source temperature 550 °C; ion spray voltage (IS) 5500 V (positive ion mode)/− 4500 V (negative ion mode); ion source gas I (GSI), gas II (GSII) and curtain gas (CUR) were set at 50, 60 and 25.0 psi, respectively; and high collision-activated dissociation (CAD). Instrument tuning and mass calibration were performed with 10 and 100 μmol/L polypropylene glycol solutions in QQQ and LIT modes, respectively. QQQ scans were acquired as MRM experiments with collision gas (nitrogen) set to medium. DP and CE for individual MRM transitions were conducted with further DP and CE optimisation [67]. A specific set of MRM transitions were monitored for each period according to the metabolites eluted within this period.

Significant differences in relative metabolite content were tested by orthogonal partial least squares discriminant analysis (OPLS-DA) with a threshold of variable important in projection (VIP) value > 1 and an absolute value of absolute value of fold change value > 2 using R software version 4.0.3 (http://www.r-project.org/).

### qRT-PCR analysis

Seven DEGs were selected for qRT-PCR analysis with actin (β-actin) as internal reference gene. Primers were designed through NCBI official website (https://www.ncbi.nlm.nih.gov/) and are listed in Additional file 1: Table S8.

The RNA extracted from *T. hispida* was used to synthesise first-strand cDNA with SweScript RT I First Strand cDNA Synthesis Kit following the manufacturer’s instructions. qRT-PCR was performed with a 2*Universal Blue SYBR Green qPCR Master Mix kit in accordance with the manufacturer’s instructions. The experimental conditions were set as follows: 40 cycles at 95 °C for 30 s (predegeneration), 95 °C for 15 s, 60 °C for 10 s and 72 °C for 30 s. The mRNA expression level of the genes was calculated with the 2^−ΔΔCt^ method. Each plant sample was analysed three times (each replicate contained three technical replicates).

## Supplementary Information

**Additional file 1 Table S1.** Changes in the fresh weight of *Tamarix hispida* seeds during germination. **Table S2.** Expression of differentially expressed genes and the detailed information of gene annotation in the seeds of *Tamarix hispida* seeds during germination. **Table S3.** Results of KEGG enrichment of differentially expressed genes in the seeds of *Tamarix hispida* during germination. **Table S4.** KEGG enrichment of differentially expressed genes in flavonoid biosynthesis (Ko00941) during seed germination and post-germination. **Table S5.** KEGG enrichment of differentially expressed genes in plant hormone signal transduction (ko04075) during seed germination and post-germination. **Table S6.** Results of GO and KEGG enrichment of differentially expressed genes in modules. **Table S7.** Differentially accumulated metabolites in stages 3, 4 and 5. **Table S8.** List of primers used for the relative quantification of gene transcripts.

**Additional file 2 Fig. S1.** Gene Ontology (GO) classification of DEGs. **Fig. S2.** Enriched KEGG pathways of differential expressed genes (DEGs) during the four adjacent stages of *Tamarix hispida* seed germination. **Fig. S3.** Enriched Gene Ontology (GO) terms of differential expressed genes (DEGs) during the six adjacent stages of *Tamarix hispida* seed germination and post- germination processes. **Fig. S4.** Analysis of DEGs in phenylpropanoid biosynthesis. **Fig. S5.** Enriched KEGG pathways and Gene Ontology (GO) terms of gene sets merging from specific stage-associated modules. **Fig. S6.** Enriched KEGG pathways and Gene Ontology (GO) terms of DEGs in purple, black and green modules. **Fig. S7.** Heatmap showing the contents of corresponding flavonoid expression at different stages.

## Data Availability

The raw sequence data from this study have been deposited in the publicly accessible National Genomics Data Center (NGDC, https://ngdc.cncb.ac.cn/) database as accession number PRJCA006871. The datasets supporting the conclusions of this article are included within the article and its additional files. The datasets used and/or analyzed during the current study are available from the authors on reasonable request (Hong Liu, liuhong@scuec.edu.cn).

## Abbreviations

GO: Gene Ontology
Nr: NCBI non-redundant
KEGG: Kyoto Encyclopedia of Genes and Genomes
Swiss-Prot: Swiss-prot protein database
DEG: Differentially expressed gene
DEM: Differentially expressed metabolite
EBG: Early biosynthetic gene
LBG: Late biosynthetic gene
AUX: Auxin
GA: Gibberellin
ABA: Abscisic acid
ETH: Ethylene
JA: Jasmonic acid
SA: Salicylic acid
WGCNA: Weighted gene co-expression network analysis
PCC: Pearson correlation coefficient
TF: Transcription factor
VIP: Variable importance in projection
PCA: Principal component analysis
qRT-PCR: Real-time quantitative reverse transcriptional PCR
