# Supplementary material for: Combined transcriptomic and metabolomic analysis reveals the potential mechanism of seed germination and young seedling growth in Tamarix hispida
Source: BMC Genomics. 2022 Feb 8;23:109. doi: 10.1186/s12864-022-08341-x (PMC8826658; doi:10.1186/s12864-022-08341-x)
Supplement: Supplementary file 2 — Additional file 2 Fig. S1. Gene Ontology (GO) classification of DEGs. Fig. S2. Enriched KEGG pathways of differential expressed genes (DEGs) during the four adjacent stages of Tamarix hispida seed germination. Fig. S3. Enriched Gene Ontology (GO) terms of differential expressed genes (DEGs) during the six adjacent stages of Tamarix hispida seed germination and post- germination processes. Fig. S4. Analysis of DEGs in phenylpropanoid biosynthesis. Fig. S5. Enriched KEGG pathways and Gene Ontology (GO) terms of gene sets merging from specific stage-associated modules. Fig. S6. Enriched KEGG pathways and Gene Ontology (GO) terms of DEGs in purple, black and green modules. Fig. S7. Heatmap showing the contents of corresponding flavonoid expression at different stages. [file 12864_2022_8341_MOESM2_ESM.docx]

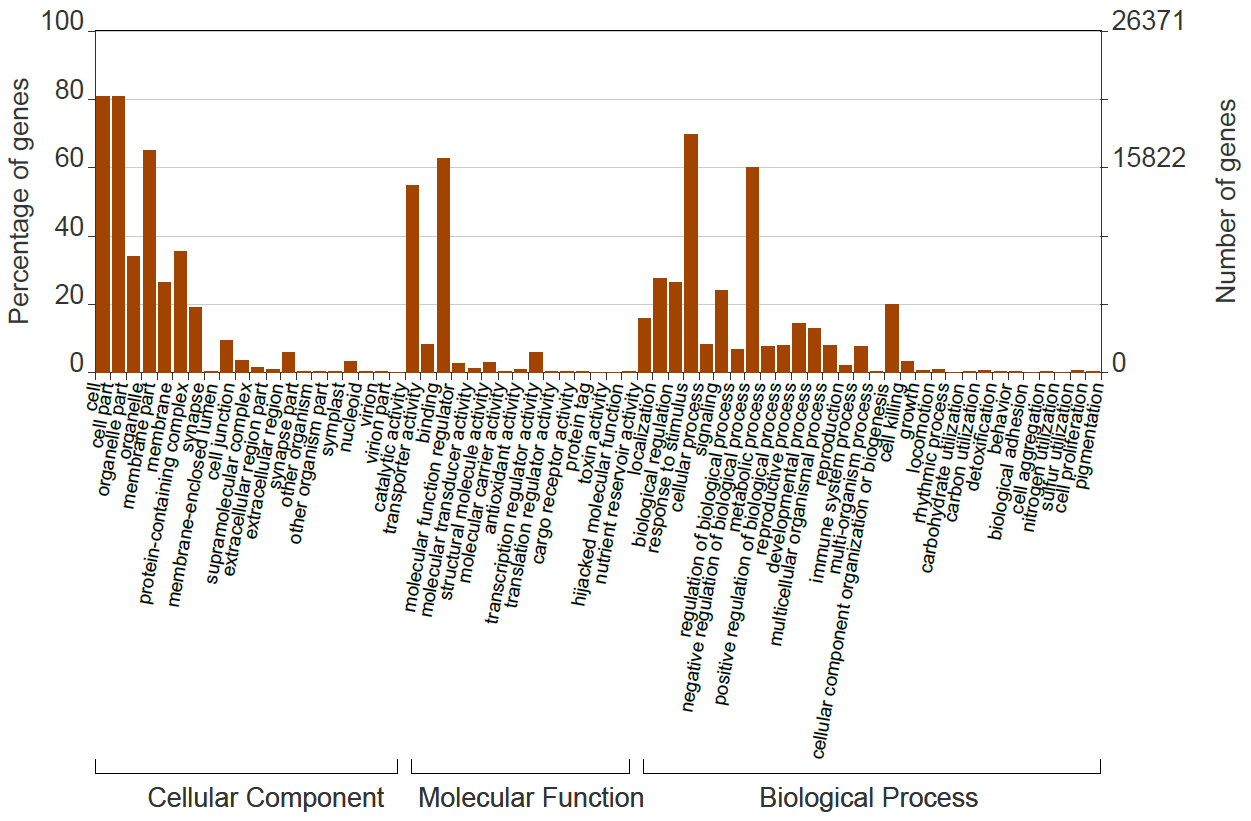


**Figure S1.** Gene Ontology (GO) classification of DEGs. The GO classification involved all annotated unigenes divided into three functional GO categories: cellular component (CC), molecular function (MF) and biological process (BP).


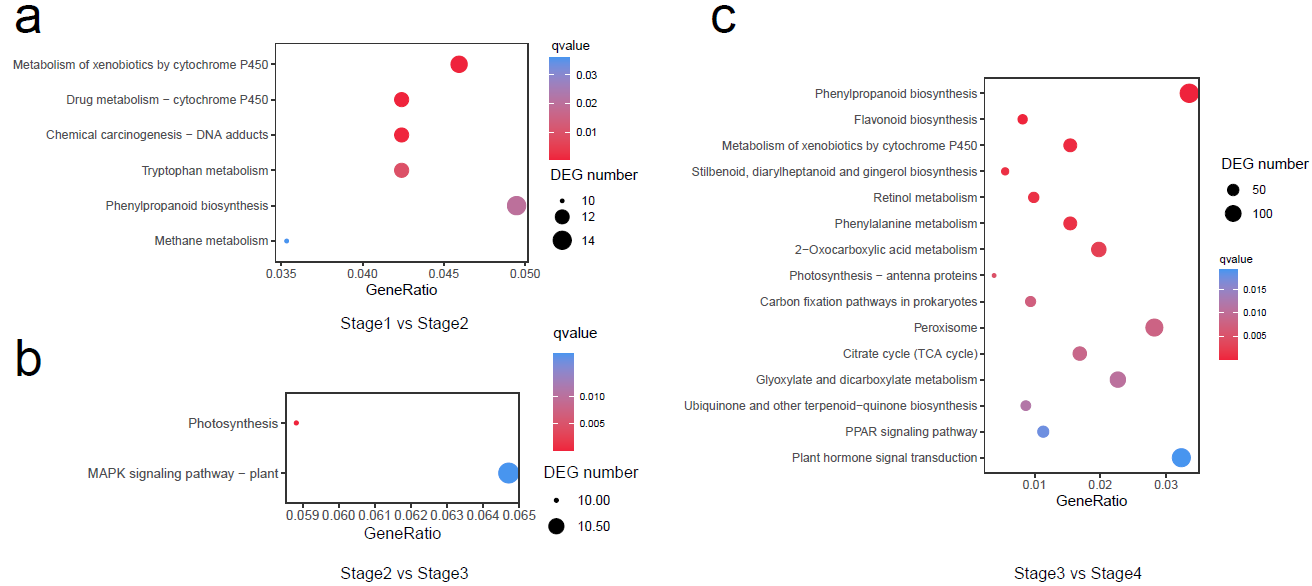


**Figure S2.** Enriched KEGG pathways of differential expressed genes (DEGs) during the four adjacent stages of *Tamarix hispida* seed germination. (a) Enriched KEGG pathways of DEGs between stages 1 and 2. (b) Enriched KEGG pathways of DEGs between stages 2 and 3. (c) Enriched KEGG pathways of DEGs between stages 3 and 4.


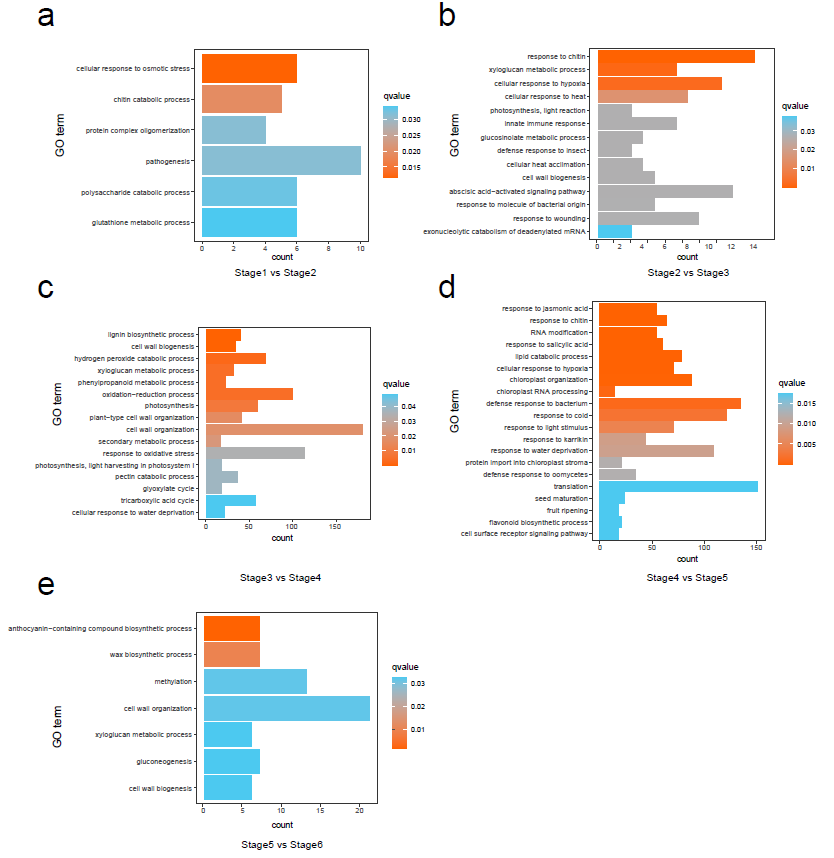


**Figure S3.** Enriched Gene Ontology (GO) terms of differential expressed genes (DEGs) during the six adjacent stages of *Tamarix hispida* seed germination and post- germination processes. (a) Enriched GO terms of DEGs between stages 1 and 2. (b) Enriched GO terms of DEGs between stages 2 and 3. (c) Enriched GO terms of DEGs between stages 3 and 4. (d) Enriched GO terms of DEGs between stages 4 and 5. (e) Enriched GO terms of DEGs between stages 5 and 6.


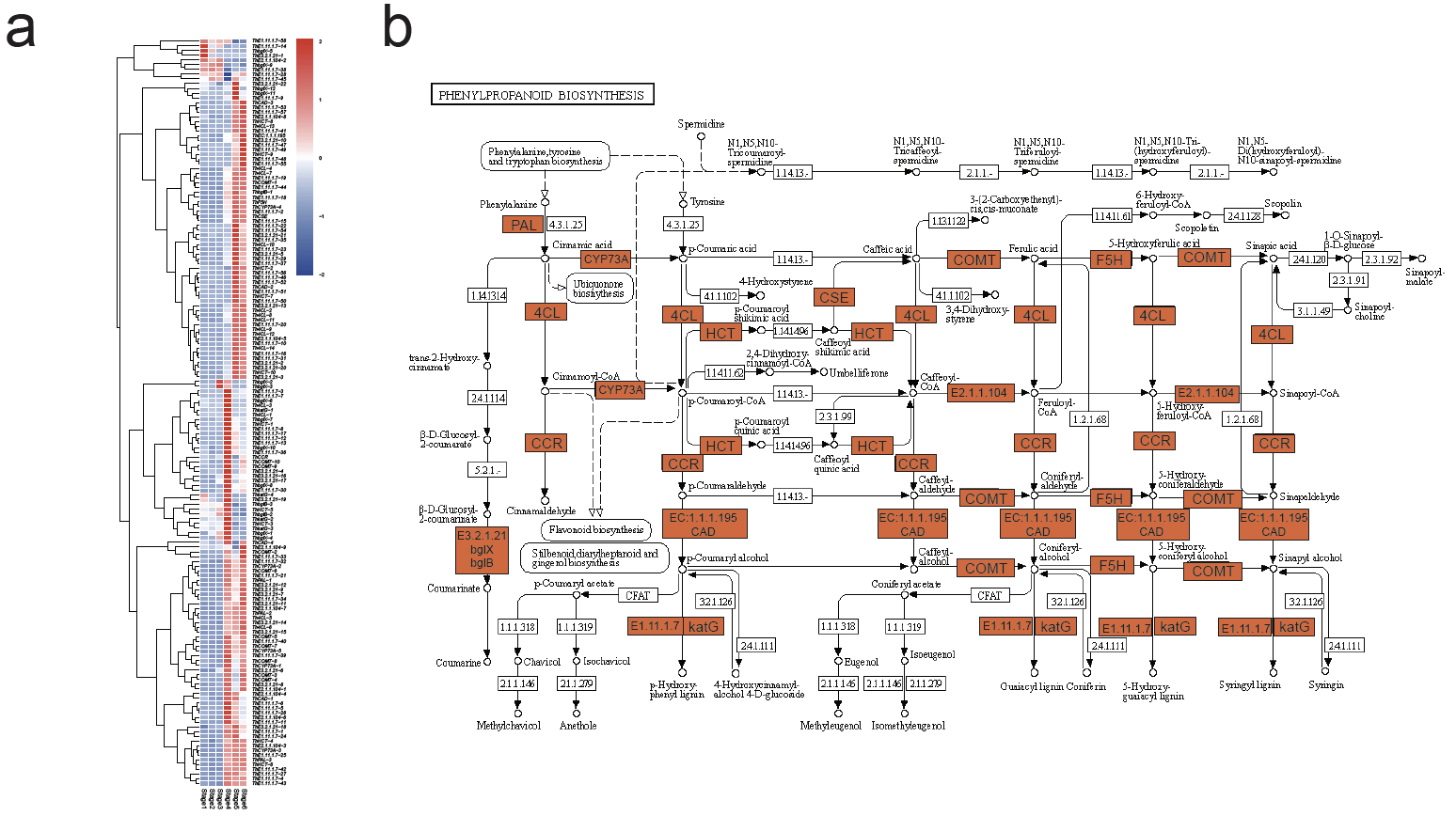


**Figure S4.** Analysis of DEGs in phenylpropanoid biosynthesis. (a) Expression pattern of DEGs involving phenylpropanoid biosynthesis in different stages. (b) Mapping of DEG enrichment in phenylpropanoid biosynthesis pathway (ko00940) [1-3]. The orange marks represent DEG enrichment.


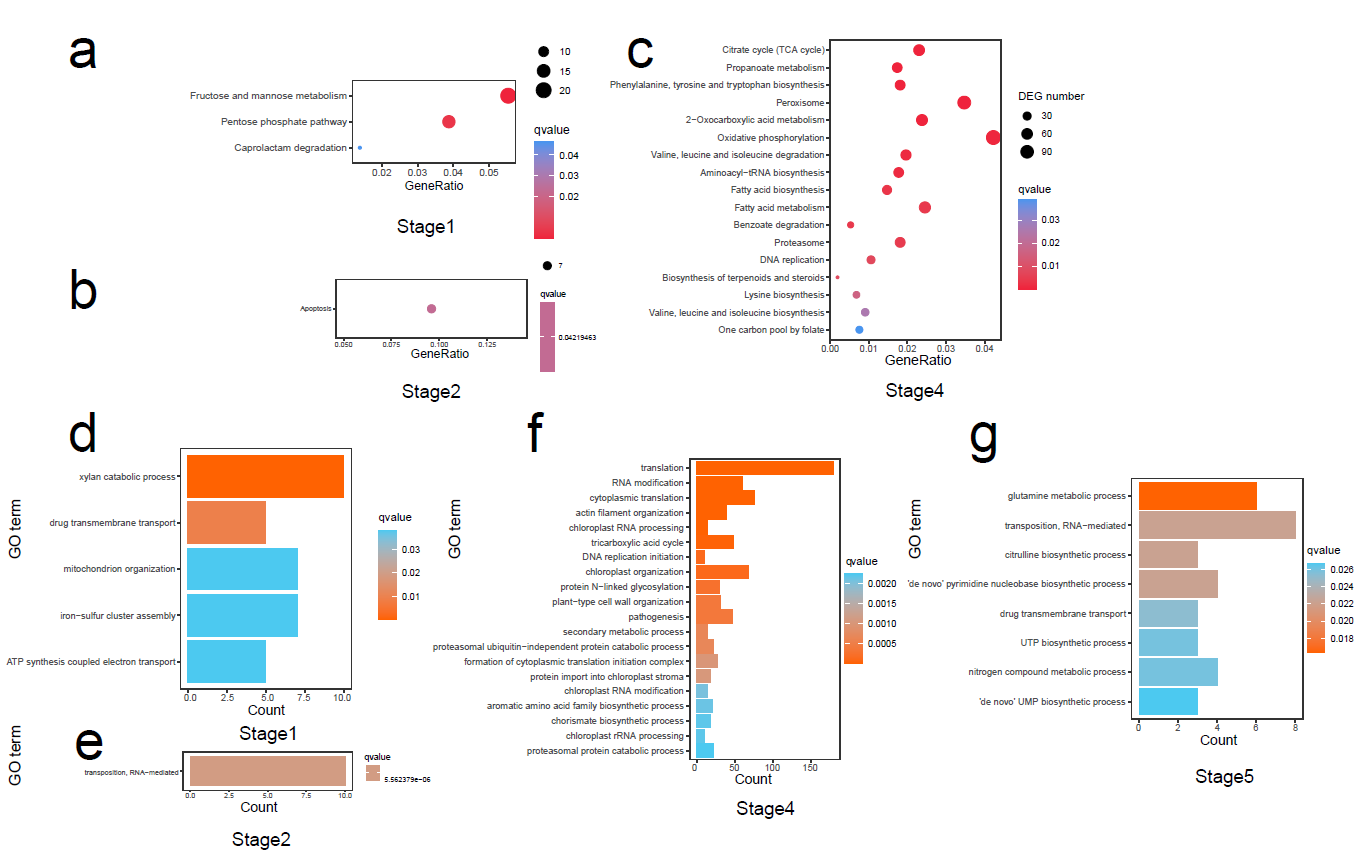


**Figure S5.** Enriched KEGG pathways and Gene Ontology (GO) terms of gene sets merging from specific stage-associated modules. (a) Enriched KEGG pathways of gene sets merging from modules (midnightblue, darkorange and skyblue) specific positively associated with stage 1. (b) Enriched KEGG pathways of gene sets merging from modules (white and cyan) specific positively associated with stage 2. (c) Enriched KEGG pathways of gene sets merging from modules (dark turquoise, salmon, black and darkred) specific positively associated with stage 4. (d) Enriched GO terms of gene sets merging from modules (midnightblue, darkorange and skyblue) specific positively associated with stage 1. (e) Enriched GO terms of gene sets merging from modules (white and cyan) specific positively associated with stage 2. (f) Enriched GO terms of gene sets merging from modules (darkturquoise, salmon, black and darkred) specific positively associated with stage 4. (g) Enriched GO terms of gene sets merging from modules (lightcyan and steelblue) specific positively associated with stage 5.


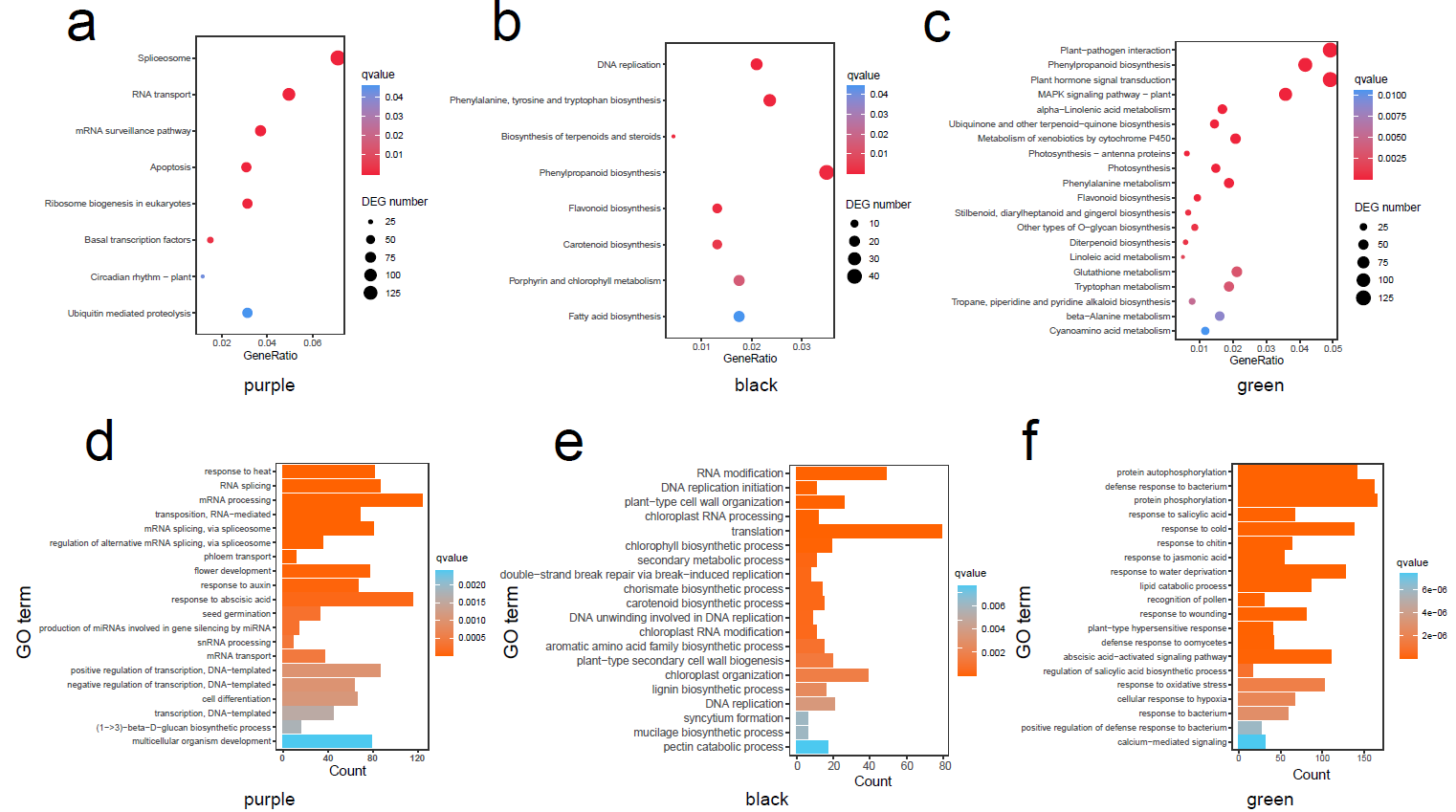


**Figure S6.** Enriched KEGG pathways and Gene Ontology (GO) terms of DEGs in purple, black and green modules. (a) Enriched KEGG pathways of DEGs in purple module. (b) Enriched KEGG pathways of DEGs in black module. (c) Enriched KEGG pathways of DEGs in green module. (d) Enriched GO terms of DEGs in purple module. (e) Enriched GO terms of DEGs in black module. (f) Enriched GO terms of DEGs in green module.


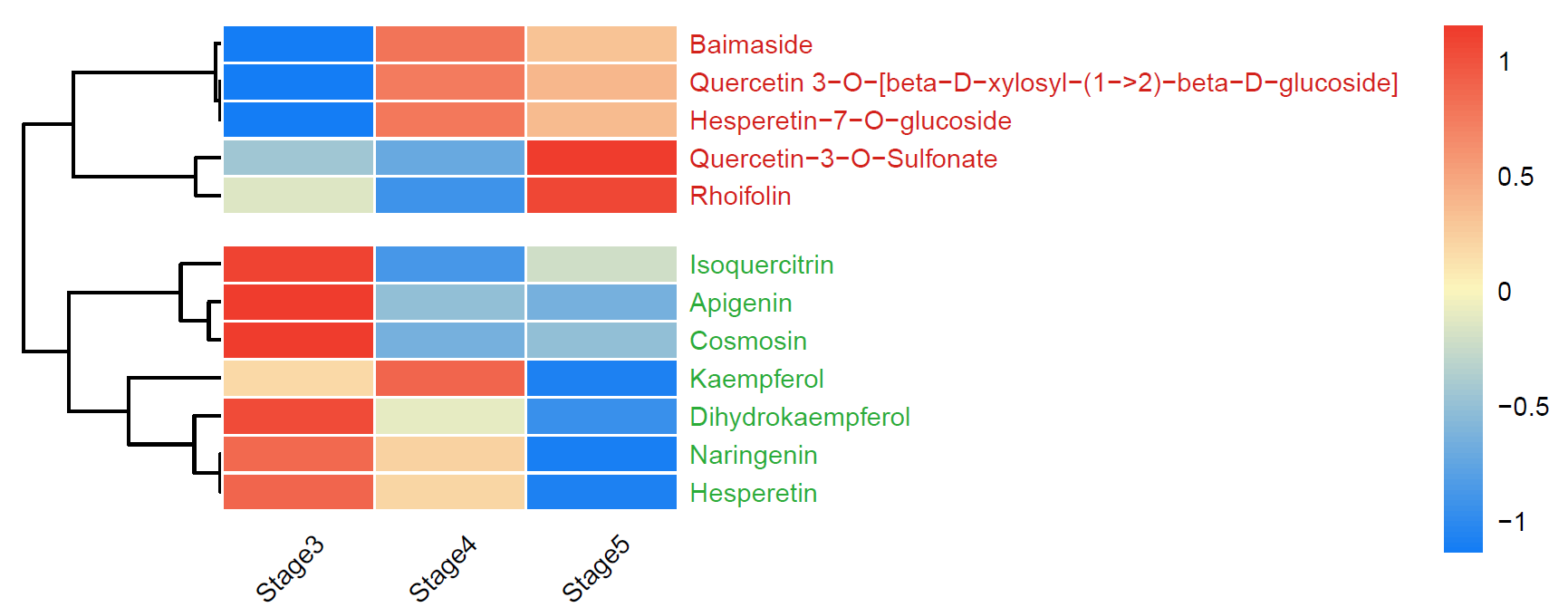


**Figure S7.** Heatmap showing the contents of corresponding flavonoid expression at different stages. Metabolic intermediates are marked in green, and end products are marked in red.

**References**

1. Kanehisa, M. and S. Goto, KEGG: kyoto encyclopedia of genes and genomes. Nucleic Acids Res, 2000. 28(1): p. 27-30.

2. Kanehisa, M., Toward understanding the origin and evolution of cellular organisms. Protein Sci, 2019. 28(11): p. 1947-1951.

3. Kanehisa, M., et al., KEGG: integrating viruses and cellular organisms. Nucleic Acids Res, 2021. 49(D1): p. D545-d551.
